# Immunoepidemiological Profiling of Onchocerciasis Patients Reveals Associations with Microfilaria Loads and Ivermectin Intake on Both Individual and Community Levels

**DOI:** 10.1371/journal.pntd.0002679

**Published:** 2014-02-20

**Authors:** Kathrin Arndts, Sabine Specht, Alexander Y. Debrah, Francesca Tamarozzi, Ute Klarmann Schulz, Sabine Mand, Linda Batsa, Alexander Kwarteng, Mark Taylor, Ohene Adjei, Coralie Martin, Laura E. Layland, Achim Hoerauf

**Affiliations:** 1 Institute of Medical Microbiology, Immunology and Parasitology (IMMIP), University Hospital Bonn, Bonn, Germany; 2 Kumasi Centre for Collaborative Research in Tropical Medicine (KCCR), Kumasi, Ghana; 3 Faculty of Allied Health Sciences and School of Medical Sciences of Kwame Nkrumah University of Science and Technology, Kumasi, Ghana; 4 Liverpool School of Tropical Medicine, Pembroke Place, Liverpool, United Kingdom; 5 Institute of Medical Biometry, Informatics and Epidemiology (IMBIE), University Hospital Bonn, Bonn, Germany; 6 UMR 7245 MCAM MNHN CNRS, Museum National d'Histoire Naturelle, Paris, France; Washington University School of Medicine, United States of America

## Abstract

Mass drug administration (MDA) programmes against *Onchocerca volvulus* use ivermectin (IVM) which targets microfilariae (MF), the worm's offspring. Most infected individuals are hyporesponsive and present regulated immune responses despite high parasite burden. Recently, with MDA programmes, the existence of amicrofilaridermic (a-MF) individuals has become apparent but little is known about their immune responses. Within this immunoepidemiological study, we compared parasitology, pathology and immune profiles in infection-free volunteers and infected individuals that were MF^+^ or a-MF. The latter stemmed from villages in either Central or Ashanti regions of Ghana which, at the time of the study, had received up to eight or only one round of MDA respectively. Interestingly, a-MF patients had fewer nodules and decreased IL-10 responses to all tested stimuli. On the other hand, this patient group displayed contrary IL-5 profiles following *in vitro* stimulation or in plasma and the dampened response in the latter correlated to reduced eosinophils and associated factors but elevated neutrophils. Furthermore, multivariable regression analysis with covariates MF, IVM or the region (Central vs. Ashanti) revealed that immune responses were associated with different covariates: whereas *O. volvulus*-specific IL-5 responses were primarily associated with MF, IL-10 secretion had a negative correlation with times of individual IVM therapy (IIT). All plasma parameters (eosinophil cationic protein, IL-5, eosinophils and neutrophils) were highly associated with MF. With regards to IL-17 secretion, although no differences were observed between the groups to filarial-specific or bystander stimuli, these responses were highly associated with the region. These data indicate that immune responses are affected by both, IIT and the rounds of IVM MDA within the community. Consequently, it appears that a lowered infection pressure due to IVM MDA may affect the immune profile of community members even if they have not regularly participated in the programmes.

## Author Summary

Onchocerciasis affects over 37 million people in poor communities. Infected individuals usually present a generalized form which is characterized by low inflammation, tightly-controlled immune responses and high parasite burden. In contrast, patients with chronic dermatitis (sowda) have reduced parasite loads and severe pathology. Current disease control is based on mass drug administration (MDA) with ivermectin (IVM) which mainly eliminates microfilariae (MF), the transmission life-stage. In hyperendemic areas, 95% of individuals are MF^+^, whereas in hypoendemic areas the rate is <30%. Recently, in areas that have been part of MDA programmes, individuals have been identified that present nodules but are amicrofilaridermic (a-MF). The following study therefore compared immunological profiles of a-MF and MF^+^ individuals and determined that only a handful of parameters significantly differed. Multivariable regression analysis revealed that immune responses were correlated to covariates MF, IVM or even the residing area (implying differing rounds of IVM MDA). For example, IL-5 responses were highly associated with MF whereas IL-17 responses were related to the region. The data imply that this “man-made” group has a distinct immune profile but also suggests that immune responses are affected by the amount of IVM intake on both the individual and community level (region).

## Introduction

Infections with *Onchocerca volvulus* commence with the transmission of larvae by biting vector hosts (black flies) [1], [2]. Female filariae create and dwell within subcutaneous nodules called onchocercomas [3], [4] whereas male worms do not take up permanent residency but migrate in the subcutaneous tissue between nodules inseminating numerous females [4]. Over an average of 10 years, females produce millions of offspring, microfilariae (MF), which primarily reside in the skin for 12–18 months [4], [5], [6]. The most severe disease manifestations are not usually induced by adult worms but elicited by the death of MF passing through the skin and corneas: this leads to various manifestations of dermatitis, and decreases in visual capacity may ultimately lead to blindness [1], [7], [8]. Through mass drug administration (MDA) programmes, cases of river blindness are becoming more scarce but despite this good news >102 million people remain at risk and the impact of dermal manifestations on daily life should not be underestimated [9], [10]. Currently, ivermectin (IVM) remains the only drug which is recommended for MDA regimes but it targets MF and has not much, if any, effect on adult worms [11]. Research on the endosymbiotic relationship between the bacteria *Wolbachia* and filariae, has revealed that doxycycline therapy targets and destroys the bacteria [12], [13]. Since *Wolbachia* are essential for the worms' fertility and survival, this treatment constitutes the only safe macrofilaricidal mechanism [5], [14].

*O. volvulus*-infected individuals who reside in areas that have received none or only a few rounds of MDA present a spectrum of disease symptoms with two polar forms, generalized onchocerciasis (GEO) or sowda. GEO or hyporesponsive individuals have palpable nodules under their skin but no strong pathology despite carrying high MF skin loads [15]. Patients with severe pathology on the other hand are termed hyperreactive or sowda [16], [17]. Histological assessment of nodules allows one to decipher between previously interrupted transmission (mainly aged worms past the time of fecundity) and females with pre-formed embryos but no developed MF [18]. Thus, despite being elicited by the same parasite, the range of clinical manifestations is quite broad and such diversity is thought to reflect the intensity and type of host immune responses to the parasite, its products, the *Wolbachia* [5] and even anti-helmintic therapy. With regards to the latter, in hyperendemic areas that have received multiple rounds of IVM, a further group of individuals have been reported. These patients have adult worms and nodules but are microfilariae negative and display little pathology. Since this type of scenario is uncommon in onchocerciasis infection without interference by IVM treatment, it is now hypothesised that they stem from MDA indicating that this patient group is in essence “man-made”.

Several studies have elucidated mechanisms and cell types that are thought to play a role in modulating immune responses in filaria-infected individuals. Amongst them are regulatory T cells (Treg), IL-10 producing Tr1 cells, alternatively activated macrophages (AAMs) and the immuno-modulatory capacity of *Wolbachia*, especially on innate cells [19], [20], [21], [22], [23], [24]. As mentioned above, hyporesponsive patients are considered to have a regulated immune system and are characterized by high levels of IL-10, Treg populations, and IgG4 [16], [17], [20], [24]. The latter is known to be induced by Treg and to be a poor inducer of antibody-dependent cell-mediated cytotoxicity (ADCC) since it cannot fix complement and binds rather weakly to effector cell Fc receptors [17], [25], [26], [27]. Since IgG4 binds to the same receptor as IgE, it has been hypothesized that elevations of this immunoglobulin prevent overt immune responses. Indeed, sowda patients portray quite the opposite picture in that they have dominant Th2 responses, high levels of IgE and eosinophilia and very few worms [28]. This is also reflected in nodule pathology [6], [29]. Most immunological studies have focused on comparing hyporeactive (MF^+^) with sowda and exposed but non-infected individuals termed endemic normals (EN). Interestingly, when compared to hyporeactive patients, EN display stronger antigen-specific proliferation and a mixed Th1/Th2 dichotomy [24], . To date however, very little is known about the final population who are amicrofilaridermic (a-MF) [33], [34]. These patients are actually a dead end for the parasite since the lack of MF limits transmission. Moreover, if current theories are correct, this population is thought to stem from repeated IVM therapy. Within this study, we took an in-depth look at filarial-specific and bystander immune responses in infection-free volunteers, MF^+^ and a-MF individuals. Patients lacking MF showed elevated numbers of neutrophils but dampened eosinophilia and associated factors in the periphery, reduced pathological parameters and IL-10 responses but elevated *O. volvulus*-specific IL-5 responses. Moreover, using multivariable regression analysis, unique immune aspects of infected individuals were found to be highly correlated with either the presence of MF or ivermectin intake on both personal and community based levels.

## Materials and Methods

### Study population and ethics statement

In 2009, we examined a cohort of 210 *O. volvulus* infected individuals that resided in 24 villages adjacent to the river Offin in Ghana (Upper and Lower Denkyira Districts in the Central Region and the Amansie Central and Adanse South Districts in the Ashanti Region). These rain forest areas are within vector range (<12 km), are hyperendemic for onchocerciasis but not other filarial infections and were not part of either OCP or APOC programmes. MDA has been implemented by the Ghanaian Ministry of Health since 2001 in Upper and Lower Denkyira districts and from 2008 in the Amansie and Adanse South areas. However, MDA compliance has not been overly effective, especially in the Upper and Lower Denkyira districts, and therefore, at the time of sampling a considerable number of people had not or not frequently taken part in IVM therapy. Men and women (18–55) in the cohort were recruited as part of the study entitled “Comparison of doxycycline alone vs doxycycline plus rifampicin in their efficacy against onchocerciasis” registered with Current Control Trials as ISRCTN68861628 (http://www.controlled-trials.com/ISRCTN68861628/hoerauf). Participants also completed a study questionnaire about their medical history which included a section about the number of times they had taken IVM and this information was checked, when possible, against reports provided by community health workers and distributors. All individuals had at least one nodule and the entire study group had, on average, an individual IVM intake (IIT) of 1.5. In the four months prior to sampling, 4 patients had taken IVM and at the time of sampling 2/4 were MF^+^. Of note, the data presented here were performed before the patients started any treatment the trial protocol is referring to. Ethical clearance, including immunological studies, was given by the Committee on Human Research Publication and Ethics at the University of Science and Technology in Kumasi, Ghana, the ethics committee at the University of Bonn, Germany and the Liverpool School of Tropical Medicine, UK. For comparison, samples were collected from 12 infection-free volunteers. These volunteers resided in non-rural areas of the same districts and are thus classified here as non-endemic normals (NEN). Written informed consent was obtained from all individuals.

### Parasitological assessment

All infected patients presented at least one palpable nodule [35]. For MF analysis, two skin biopsies of 1–3 mg were taken from the buttocks using a corneoscleral (Holth) punch (Koch, Hamburg, Germany). Each biopsy was immersed in 100 µl of 0.9% NaCl solution in a well of a microtiter plate (Nunc, Roskilde, Denmark). The skin biopsies were incubated overnight at room temperature to allow MF to emerge. The solution was then transferred onto a slide for microscopic examination [14], [36]. The biopsies were weighed using a Sartorius electronic balance (Göttingen, Germany) and MF load was calculated per mg skin. Blood smears from all patients were screened for their cellular compositions and malaria infections using standard Giemsa staining protocols. The presence of further helminth infections were assessed using standard diagnostic tests on stool and urine samples [37].

### Antigens and antibodies

A soluble extract of *Onchocerca volvulus* worm antigen (OV) was prepared as previously described [20] and extracts of *Brugia malayi* female worms were prepared from infected jirds that were or were not treated with tetracycline (BmFE and BmFEtet, [38]). Recombinant peptidoglycan-associated lipoprotein (wPAL) and *Wolbachia* surface protein (WSP) were prepared as previously described [38], [39]. LPS (*Serratia marescens*) was obtained from Sigma-Aldrich (Taufkirchen, Germany) and tuberculin purified protein derivative (PPD) from Statens Serum Institute (Copenhagen, Denmark). Anti-CD3 and anti-CD28 antibodies were purchased from eBiosciences (Frankfurt, Germany). Polymyxin B sulphate salt (P4932) and secondary antibodies for IgG1-4 were obtained from Sigma-Aldrich, whereas that for IgE was purchased from Southern Biotech (Birmingham, USA).

### Assessment of *O. volvulus*-specific IgE and IgG subclasses

Individual plasma samples were screened for the content of filarial-specific IgE and IgG1-4. In brief, 96-well polysorb plates (Nunc, Roskilde, Denmark) were coated overnight at 4°C with 5 µg/ml *O. volvulus* extract in PBS (pH 9.6). Plates were washed 3 times in 0.05% Tween/PBS (pH 7.2) and once with PBS alone. Plates were then blocked with 200 µl/well of 1% BSA/PBS for two hours at room temperature. After an additional washing step, 50 µl/well of diluted plasma was added in triplicate (1∶500 for IgG1-4 and 1∶20 for IgE) and incubated overnight at 4°C. After washing, 50 µl/well of biotinylated secondary antibodies were added for two hours at room temperature, IgG1 (1∶1,000), IgG2 (1∶15,000), IgG3 (1∶4,000), IgG4 (1∶15,000); IgE (1∶1,000). Following the next wash, 50 µl/well streptavidin-peroxidase (Roche Diagnostics, Mannheim, Germany; 1∶5,000) was incubated for 45 minutes at room temperature. After the final wash, 50 µl/well of substrate solution containing TMB (tetramethylbenzidine, Sigma-Aldrich) was used and the reaction was stopped with 25 µl/well 2N H_2_SO_4_ (Sigma-Aldrich). The plates were measured using the SpectraMAX ELISA reader (Molecular Devices, Sunyvale, U.S.) with wavelength correction (450 nm and 570 nm). Data were analyzed with SOFTmax Pro 3.0 software. Pooled plasma from 15 patients was used to generate standard calibration curves which were assigned arbitrary units. This standard was used for each measurement of anti-filarial antibodies. In plasma from healthy European donors *O. volvulus*-specific IgG1-4 and IgE were not detectable (data not shown).

### PBMC preparation and *in vitro* cell cultures

PBMCs were isolated as previously described [37]. Plasma samples were frozen at −20°C until further required. Cell suspensions were washed twice with sterile PBS (8 min at 400× g room temperature) and re-suspended in RPMI 1640 medium (PAA, Linz, Austria), supplemented with 10% FCS, 2 mM L-glutamine, 50 µg/ml penicillin/streptomycin and 50 µg/ml gentamicin (all PAA) before determining the cell concentration with trypan blue (Sigma-Aldrich). Thereafter, 2×10^5^ PBMCs/well were plated onto 96-well plates (U-shaped, Greiner Bio-One, Frickenhausen, Germany). PBMCs were then left either un-stimulated or stimulated in triplicate with the following stimuli: OV (5 µg/ml), BmFE (5 µg/ml), BmFEtet (5 µg/ml), anti-CD3/anti-CD28 (10 µg/ml and 2.5 µg/ml respectively), LPS (50 ng/ml), PPD (10 µg/ml), wPAL and WSP (both at 5 µg/ml in combination with 50 µg/ml of Polymyxin B). Cultures containing Polymyxin B alone elicited only background cytokine responses (data not shown). Cultures were incubated for 72 hours at 37°C in 5% CO_2_. Supernatants were collected and frozen until further use.

### Cytokine measurements

Culture supernatants from stimulated PBMCs were thawed on ice and analyzed for the content of IL-5, IL-6, IL-10, IL-13, IL-17, IFN-γ and TNF using R&D Duo sets (R&D Systems, Wiesbaden-Nordenstadt, Germany) in accordance with the manufacturer's instructions. The plates were measured and analyzed as mentioned above.

### Analysis of IL-5 and ECP levels in plasma samples

Undiluted plasma samples from infected patients were analyzed for the content of IL-5 (Quantikine, R&D Systems) and eosinophil cationic protein (ECP) (Aviscera Bioscience, Inc, Santa Clara, USA) according to the manufacturer's instructions. The plates were measured and analyzed as mentioned above.

### Statistical analysis

Statistical analyses were performed using the software SPSS (IBM SPSS Statistics 20; Armonk, NY), the PRISM 5 programme (GraphPad Software, Inc., La Jolla, USA) and SAS version 9.2 (SAS Institute Inc. Cary, NC, USA). Since most of the variables did not show a normal distribution, the following tests were chosen: to compare three groups a Kruskal-Wallis-test was performed and, if significant, followed by a Mann-Whitney–U test for a further comparison of the groups; for comparisons of two binary variables the Fisher's exact test and for comparisons of continuous parameters the Spearman correlation was used. The immune response data were also assessed using a generalized linear model. Initially, parameters including age, gender, nodules and co-infections were included as covariates but the relevant associations were revealed with regional, IVM and MF related parameters. Therefore, a more stringent analysis was conducted using the covariates “times of individual IVM therapy (IIT)”, “IVM intake within the last 12 months”, “MF-positivity” and “microfilarial density”. In addition, we included a regional covariate, “Central∶Ashanti”, since at the time of sampling, villages in the Central region had had 7 years of MDA whereas those in the Ashanti region had only had programmes for 1 year. This covariate consisted of 14 villages (82 individuals) from the “Central” region and 10 villages (128 individuals) from the “Ashanti” region. For this model continuous variables were rank-transformed. If more than one covariate was below p<0.1 following univariable analysis, a further multivariable stage was conducted (p<0.05).

## Results

### Clinical evaluation of *Onchocerca volvulus* infected patients

**Figure 1:**
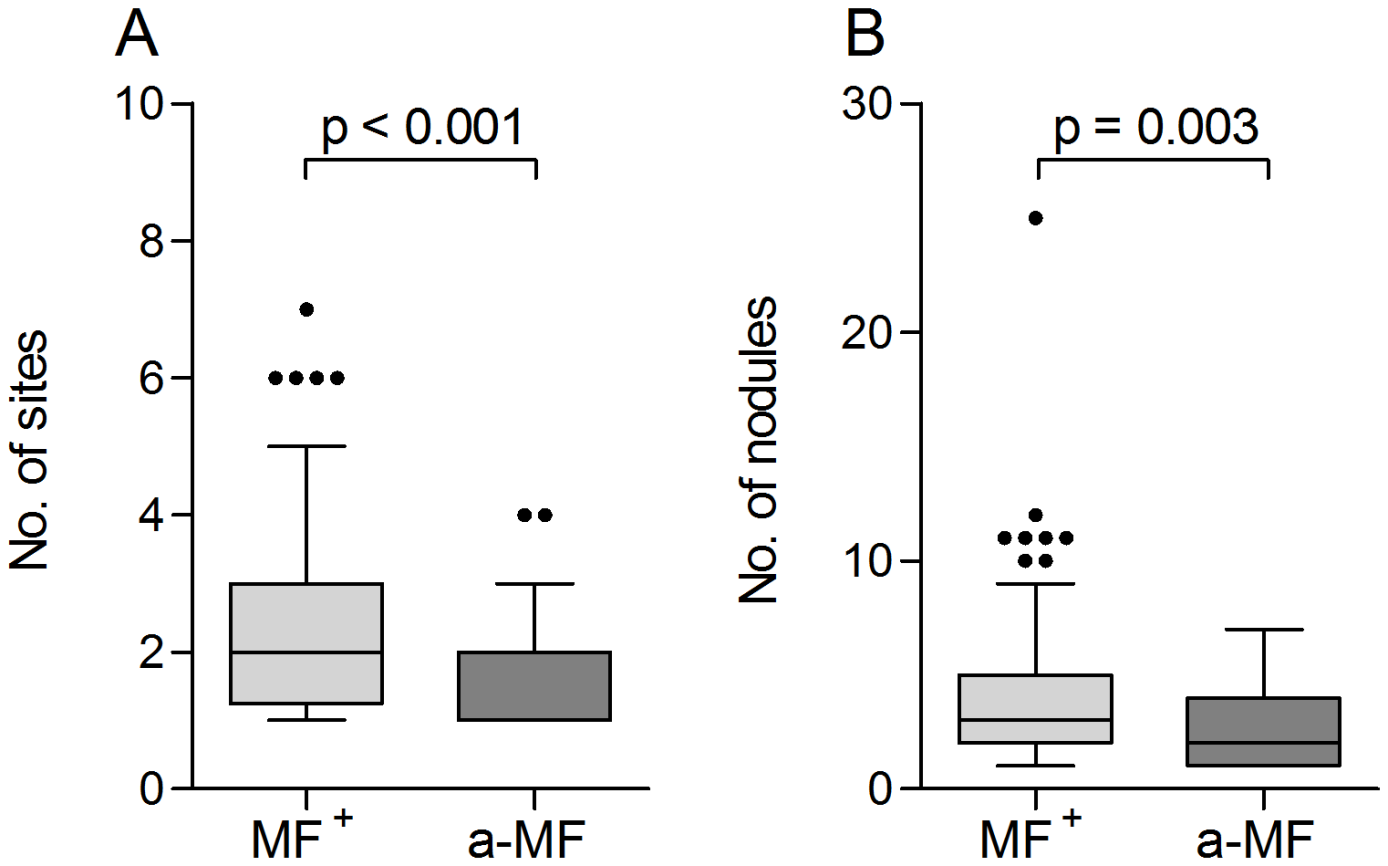
*O.volvulus* infections are characterized by the presence of nodules in the skin. MF^+^ (n = 164) and amicrofilaridermic (a-MF, n = 46) individuals were examined for the number of sites (A) and the number of nodules (B) by palpation. Graphs show box whiskers with median, interquartile ranges and outliers. As indicated, statistical significances between the groups were obtained using Mann-Whitney tests.

Within the study population described here, 22% of the infected individuals were amicrofilaridermic despite all presenting at least one nodule (which constituted an inclusion criterion for the study). 164 individuals were MF^+^ with a range from 0.06–330.81 MF/mg skin. With regards to further worm infections 8.5% MF^+^ (14/164) and 8.7% a-MF (4/46) individuals were co-infected with either hookworms (n = 13), *Schistosoma mansoni* (n = 2) or *S. haematobium* (n = 4). We determined whether the absence of MF was associated with the number of nodules and sites since these parameters reflect the amount and tissue distribution of adult worms. Upon comparison with a-MF individuals, microfilaridermic patients displayed significantly more sites and nodules (Figures 1A and B respectively). In addition, the amount of microfilariae positively correlated with both of these parameters (MF and nodules: r = 0.231, p = 0.001 and MF and sites: r = 0.317, p<0.001).

### Filarial-specific antibody levels remain unaltered in a-MF individuals

**Figure 2:**
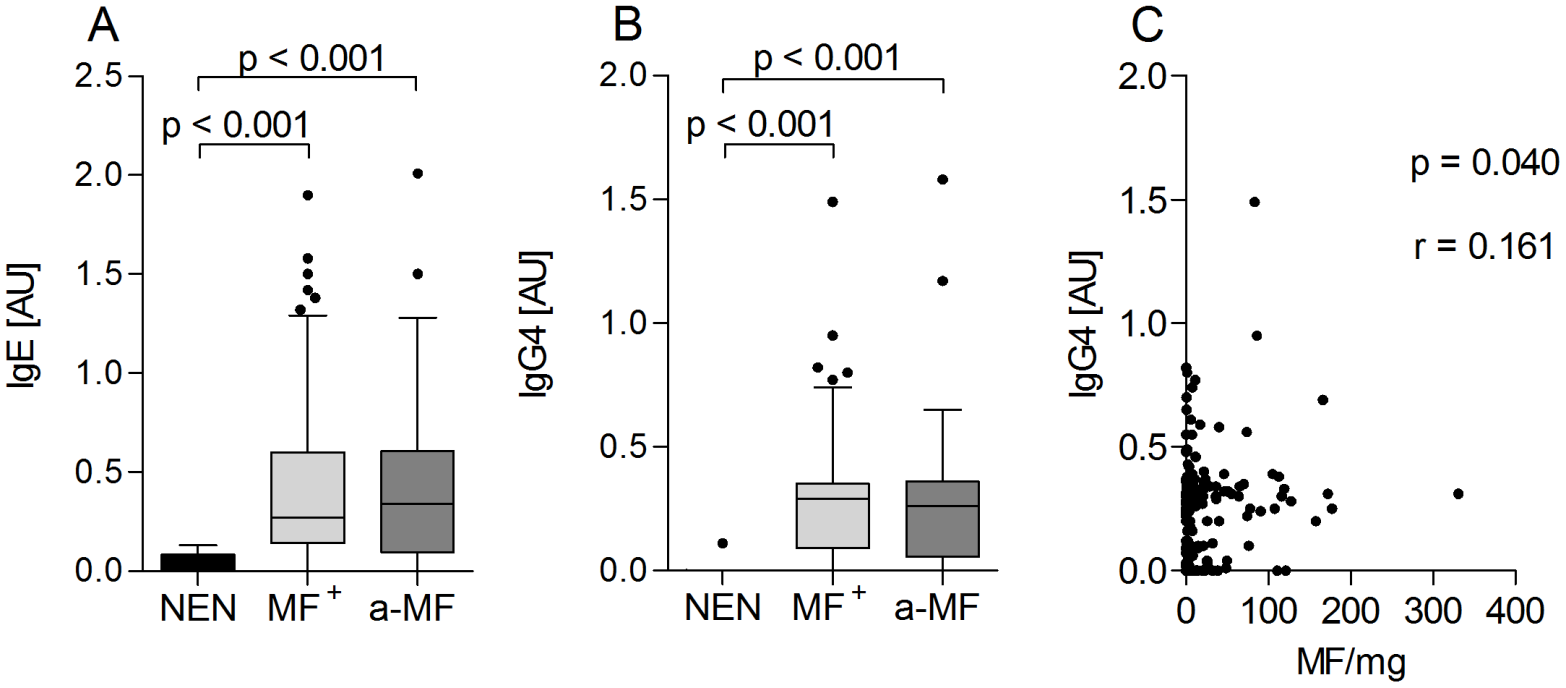
Lack of dermal residing MF does not alter filarial-specific antibody profiles. Plasma samples from all participants (NEN, MF^+^ and a-MF) were investigated for the presence of filarial-specific Igs by ELISA. Plates were coated overnight with 5 µg/ml *O. volvulus* worm extract. Thereafter, levels of IgE (A) and IgG4 (B) were detected in all individual samples diluted 1∶20 and 1∶500 respectively. Data are depicted as absolute values expressed in arbitrary units [AU]. Graphs show box whiskers with median, interquartile ranges and outliers. Statistical significances between the indicated groups were obtained after Kruskal-Wallis and Mann-Whitney tests. Data in (C) show the correlation of dermal MF with the amount of filarial-specific IgG4; determined using the Spearman correlation test.

Hyporesponsive individuals generally display increased levels of filarial-specific IgG4 [7], [17], [40]. Thus, we compared *O. volvulus*-specific Ig profiles in NEN, MF and a-MF individuals and as expected filarial-specific antibody levels were significantly increased in infected individuals (Table 1). However, when comparing Ig levels in the two infected populations, no differences could be observed in any of the measured parameters especially IgE (Figure 2A) and IgG4 (Figure 2B). Moreover, the ratio of IgG4/IgE was also not significantly altered between both groups (date not shown). Of note, MF load in MF^+^ individuals positively correlated to some IgG subclasses (IgG4: r = 0.161, p = 0.040 (Figure 2C), IgG1: r = 0.171, p = 0.029 and IgG3; r = 0.165, p = 0.036 (data not shown).

**Table 1:** *O. volvulus*-specific Ig levels of study participants shown as median and range.

	NEN (n = 12)	MF^+^ (n = 164)	a-MF (n = 46)
IgG1 [AU]	0.00 [0.00, 0.06]	0.25 [0.00, 9.72]	0.22 [0.00, 2.35]
IgG2 [AU]	0.00 [0.00, 2.70]	0.24 [0.07, 1.52]	0.28 [0.08, 1.26]
IgG3 [AU]	0.00 [0.00, 0.09]	0.17 [0.00, 3.27]	0.18 [0.00, 2.59]
IgG4 [AU]	0.00 [0.00, 0.11]	0.29 [0.00, 1.49]	0.26 [0.00, 1.58]
IgE [AU]	0.03 [0.00, 0.13]	0.27 [0.00, 1.90]	0.34 [0.00, 2.01]

Plasma samples from all participants (NEN, MF^+^ and a-MF) were investigated for the presence of *O. volvulus*-specific Igs by ELISA. Data are depicted as absolute values expressed in arbitrary units [AU]. Values show median and range.

### Elevated eosinophils and associated factors in MF^+^ individuals

**Figure 3:**
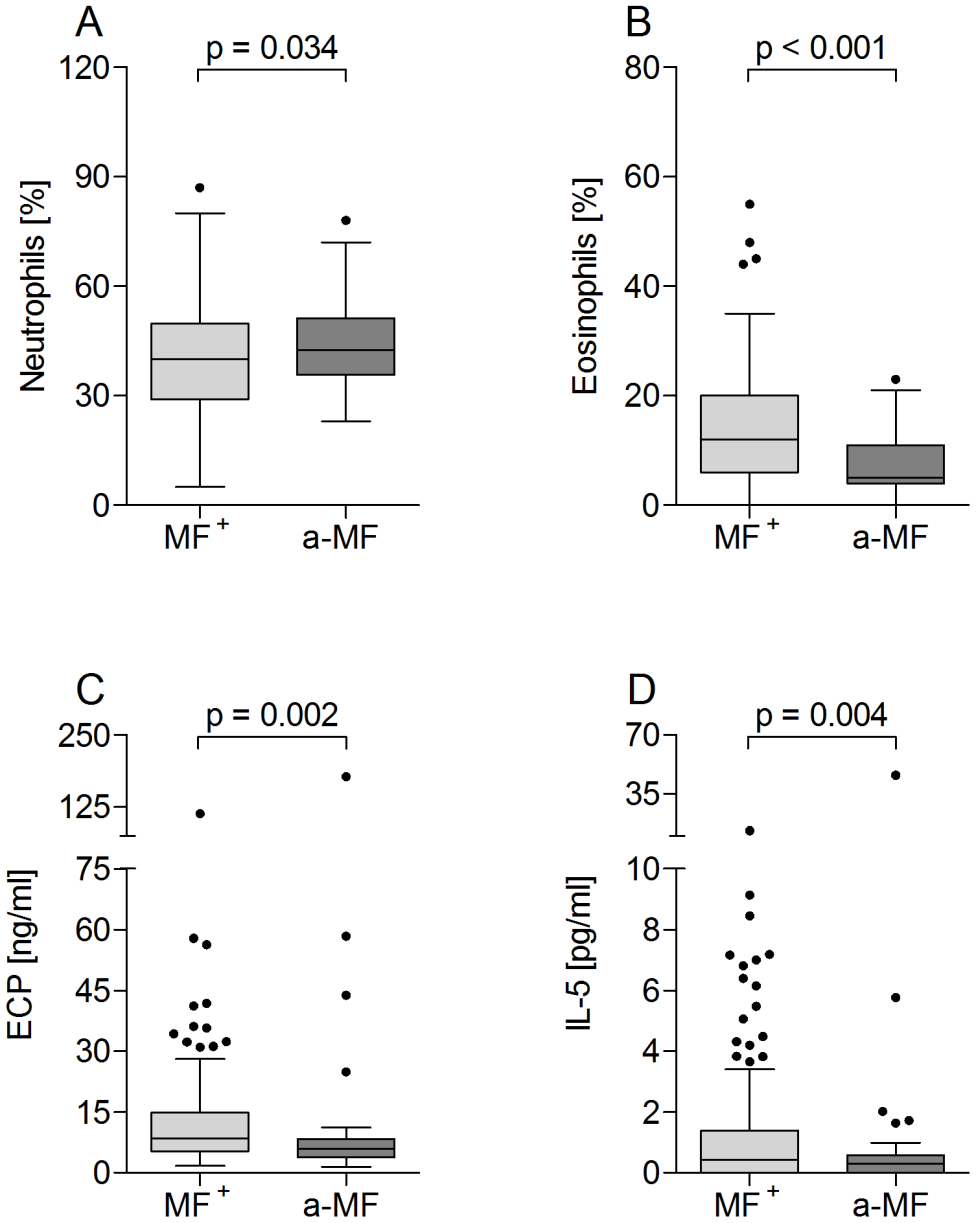
Decreased levels of eosinophils and eosinophil-associated proteins in a-MF patients. *O. volvulus* infected participants were investigated for the percentages of neutrophils (A) and eosinophils (B) using standard Giemsa stained blood smears. In addition, the presence of ECP (C) and IL-5 (D) were determined in plasma samples by ELISA. Graphs show box whiskers with median, interquartile ranges and outliers. Statistical significances between the indicated groups were obtained after Mann-Whitney tests.

**Figure 4:**
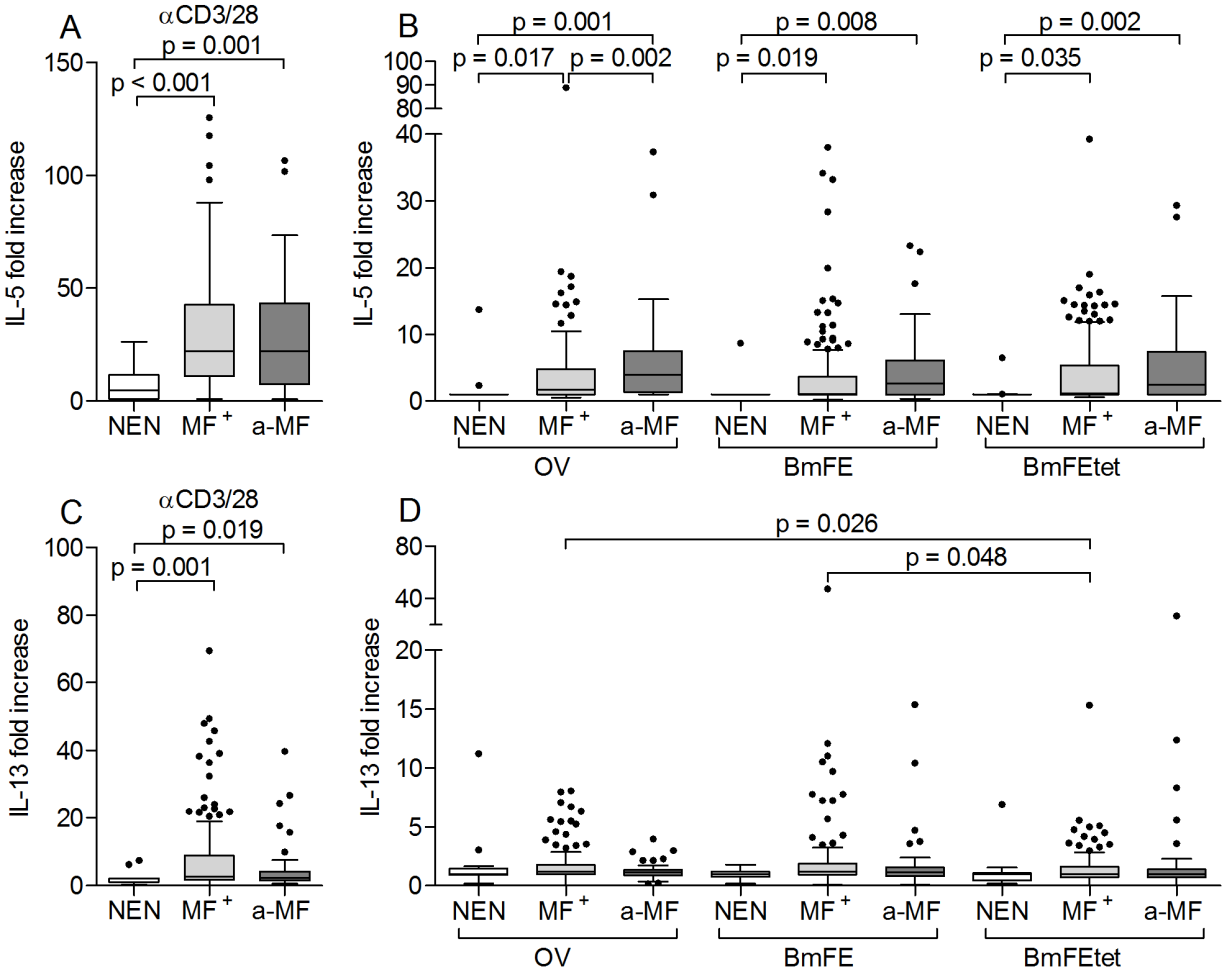
Elevated filarial-specific IL-5 production is associated with MF status. Isolated PBMCs (2×10^5^/well) from NEN or *O. volvulus* infected MF^+^ or a-MF patients were stimulated with either anti-CD3/anti-CD28 (10 µg/ml/2.5 µg/ml) (A and C), an *O. volvulus* extract (OV, 5 µg/ml), a *B. malayi* female extract (BmFE, 5 µg/ml) or an extract from *B. malayi* females worms from infected animals that were depleted of *Wolbachia* through tetracycline treatment (BmFEtet, 5 µg/ml) (B and D) for 72 hours. Thereafter, levels of IL-5 (A and B) and IL-13 (C and D) were measured in the culture supernatants via ELISA. Data are plotted as fold increase over unstimulated controls. Graphs show box whiskers with median, interquartile ranges and outliers. Statistical significances between the indicated groups were obtained after Kruskal-Wallis and Mann-Whitney tests.

Eosinophilia is a common characteristic of helminth infections and the balance between eosinophils and neutrophils has been shown to be correlated with the survival of cattle filaria, *Onchocerca ochengi* [41]. To observe whether a-MF individuals displayed altered peripheral cell differentiation, blood smears were assessed for neutrophils (Figure 3A) and eosinophils (Figure 3B), macrophages and lymphocytes (data not shown). With regards to macrophages and lymphocytes, no differences were observed between the two groups. a-MF patients did however present significantly higher numbers of neutrophils (Figure 3A) but less eosinophils (Figure 3B) which were negatively correlated with one another (Figure S1A, p>0.001; r = −0.511). Moreover, in infected patients, levels of eosinophils were positively correlated with MF load (Figure S1B), which was not the case with neutrophils (Figure S1C). Further indications of elevated eosinophil activity in MF^+^ individuals was reflected in their increased amounts of plasma bound ECP (Figure 3C), a ribonuclease superfamily member that has helmintho-toxic properties [42]. Activated eosinophils are not only drawn to IL-5 but produce it themselves and plasma levels of this cytokine were also significantly elevated in MF^+^ patients (Figure 3D). Next, we analyzed levels of IL-5 and another Th2 cytokine, IL-13, in culture supernatants of PBMCs that were stimulated with either αCD3/αCD28 (Figures 4A and C), or filarial antigen preparations (Figures 4B and D). Here, three different antigen preparations were employed: OV (a preparation of adult *O. volvulus* worms), BmFE (an extract of *B. malayi* female adult worms) and BmFEtet (female *B. malayi* adults that had been harvested from infected animals that had been treated with a tetracycline). Since the administration of such antibiotics leads to the depletion of *Wolbachia*, the latter antigen was used to determine whether the absence of this bacteria altered immune responses [38], [43], [44]. All infected patients showed elevated IL-5 and IL-13 responses when stimulated with αCD3/αCD28 but no differences were observed between the two infected groups (Figure 4A and C respectively). In contrast to the situation found in plasma, PBMCs from a-MF individuals produced significantly higher amounts of IL-5 when exposed to an OV extract (Figure 4B). Interestingly, this significance was lost when the antigen extract stemmed from a related filarial species (Figure 4B). Filarial-specific IL-13 responses were low and no differences were observed between MF^+^ and a-MF patients with any of the tested filarial antigens. However, PBMCs from MF^+^ individuals reacted better to OV and BmFE than BmFEtet indicating that either the absence of *Wolbachia* reduced this Th2 response, or that tetracycline treatment also reduced bona fide filarial antigens that contained T cell epitopes (Figure 4D).

### Elevated levels of IL-10 in patently infected patients

**Figure 5:**
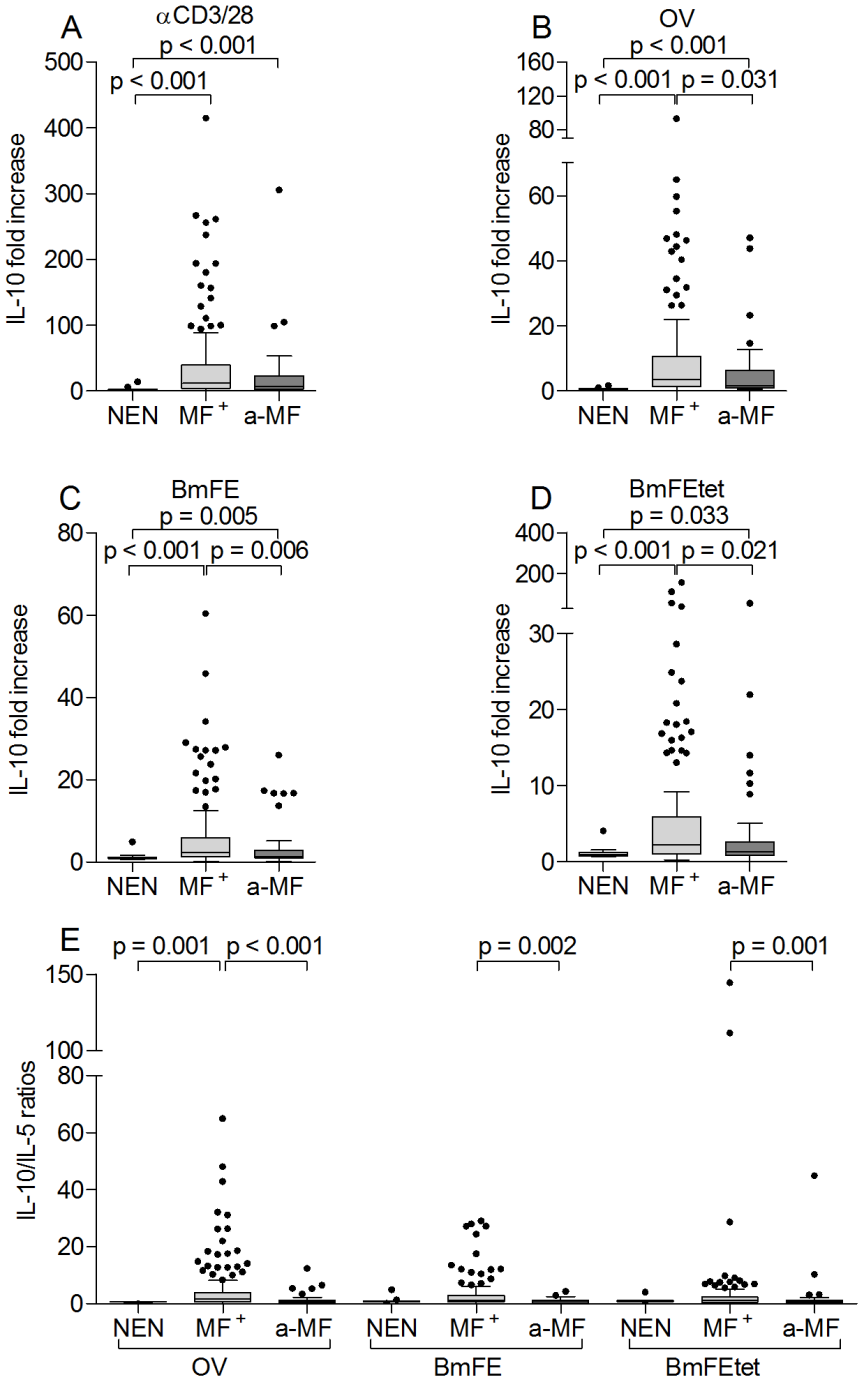
Elevated IL-10 levels in microfilaridermic patients. Isolated PBMCs (2×10^5^/well) from NEN or *O. volvulus* infected MF^+^ or a-MF patients were stimulated with either anti-CD3/anti-CD28 (A), OV (B), BmFE extract (C) or BmFEtet extract (D) for 72 hours. Thereafter, levels of IL-10 were measured in the culture supernatants via ELISA. (E) shows the ratios of IL-10/IL-5 for each stimuli. Data are plotted as fold increase over unstimulated controls. Graphs show box whiskers with median, interquartile ranges and outliers. Statistical significances between the indicated groups were obtained after Kruskal-Wallis and Mann-Whitney tests.

High levels of IL-10 have been described in onchocerciasis patients presenting the hyporeactive form of infection [7], [16], [17], [24], [45]. To analyze whether IL-10 responses were altered in a-MF individuals, isolated PBMCs were stimulated with either αCD3/αCD28 (Figure 5A) or filarial antigen preparations (Figures 5B–D). As depicted in Figure 5, all infected patients produced significantly higher amounts of IL-10 when compared to responses of PBMCs isolated from NEN. Moreover, when compared to a-MF patients, PBMCs from MF^+^ individuals had significantly stronger IL-10 responses upon exposure to all three filarial extracts (Figures 5B–D). These dominant IL-10 profiles in MF^+^ individuals were further emphasized after assessing the ratio of IL-10/IL-5 levels, which were significantly higher in MF^+^ than in a-MF patients (Figure 5E). Alongside Th2 cytokines and IL-10 (Figures 4 and 5 respectively), we also measured levels of IFN-γ and IL-17 (Figure S2). Here, no significant differences could be observed between the groups and this was independent of the applied stimulus: αCD3/αCD28 (Figures S2A and E) or filarial extracts (Figures S2B–D, F–H). In addition, we also screened immune profiles to bystander antigens such as PPD (Figure S3) or LPS (Figures S4). When compared to NEN, IL-10 responses became elevated regardless of the tested stimuli (Figures S3G and S4G). Moreover, PPD stimulated cell cultures from a-MF patients contained significantly less IL-10 when compared to responses from MF^+^ individuals (Figure S3G). Interestingly, no significant differences in Th-related cytokines were detected between the infected groups upon triggering cells with LPS (Figure S4).

### Filarial-specific up-regulation of innate cytokines in infected individuals

**Figure 6:**
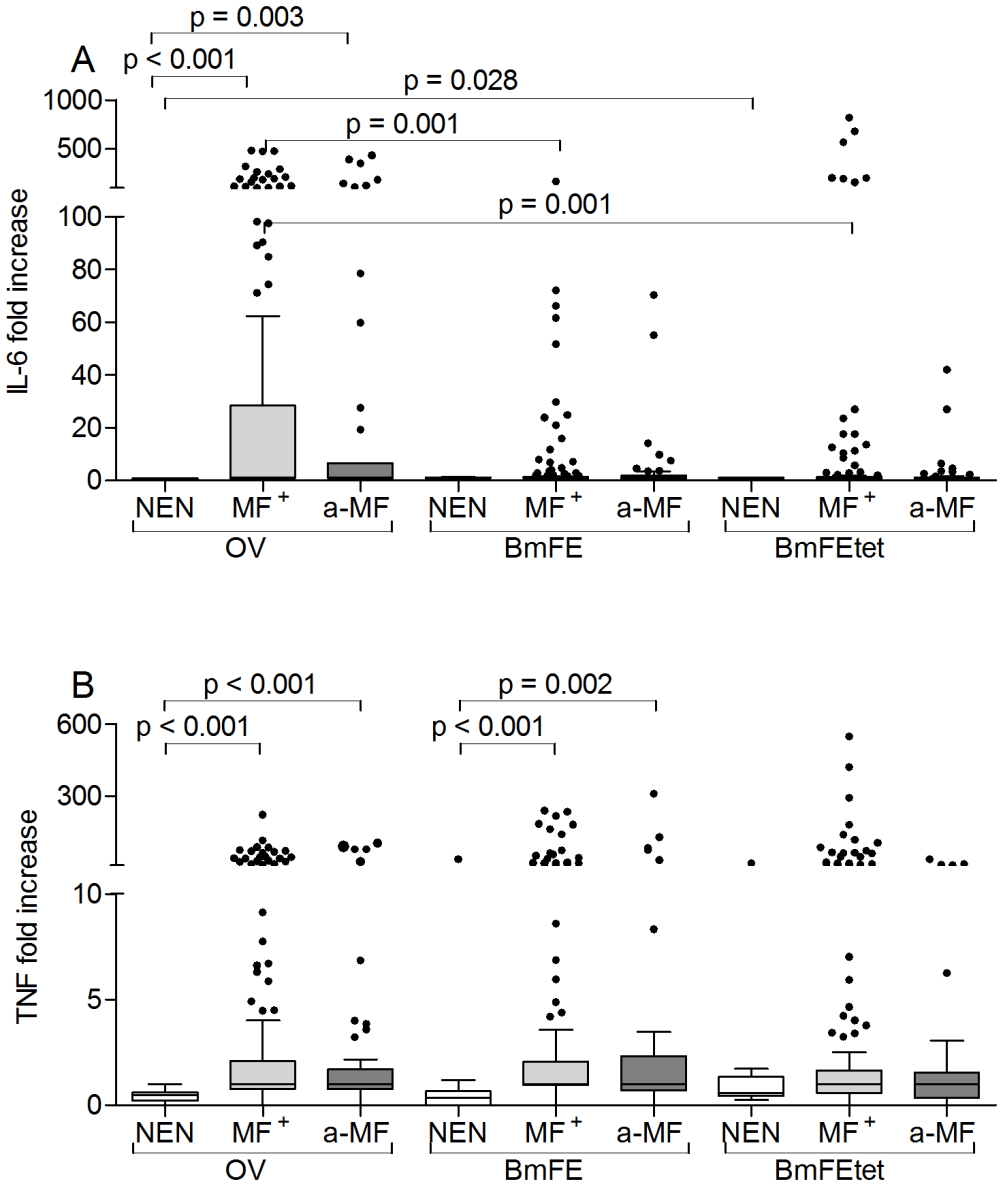
Increased *O. volvulus*-specific IL-6 and TNF responses in infected individuals. Isolated PBMCs (2×10^5^/well) from NEN or *O. volvulus* infected MF^+^ or a-MF patients were stimulated with either OV extract, BmFE extract or BmFEtet extract. After 72 hours, levels of IL-6 (A) and TNF (B) were measured in the culture supernatants via ELISA. Data are plotted as fold increase over unstimulated controls. Graphs show box whiskers with median, interquartile ranges and outliers. Statistical significances between the indicated groups were obtained after Kruskal-Wallis and Mann-Whitney tests.

**Figure 7:**
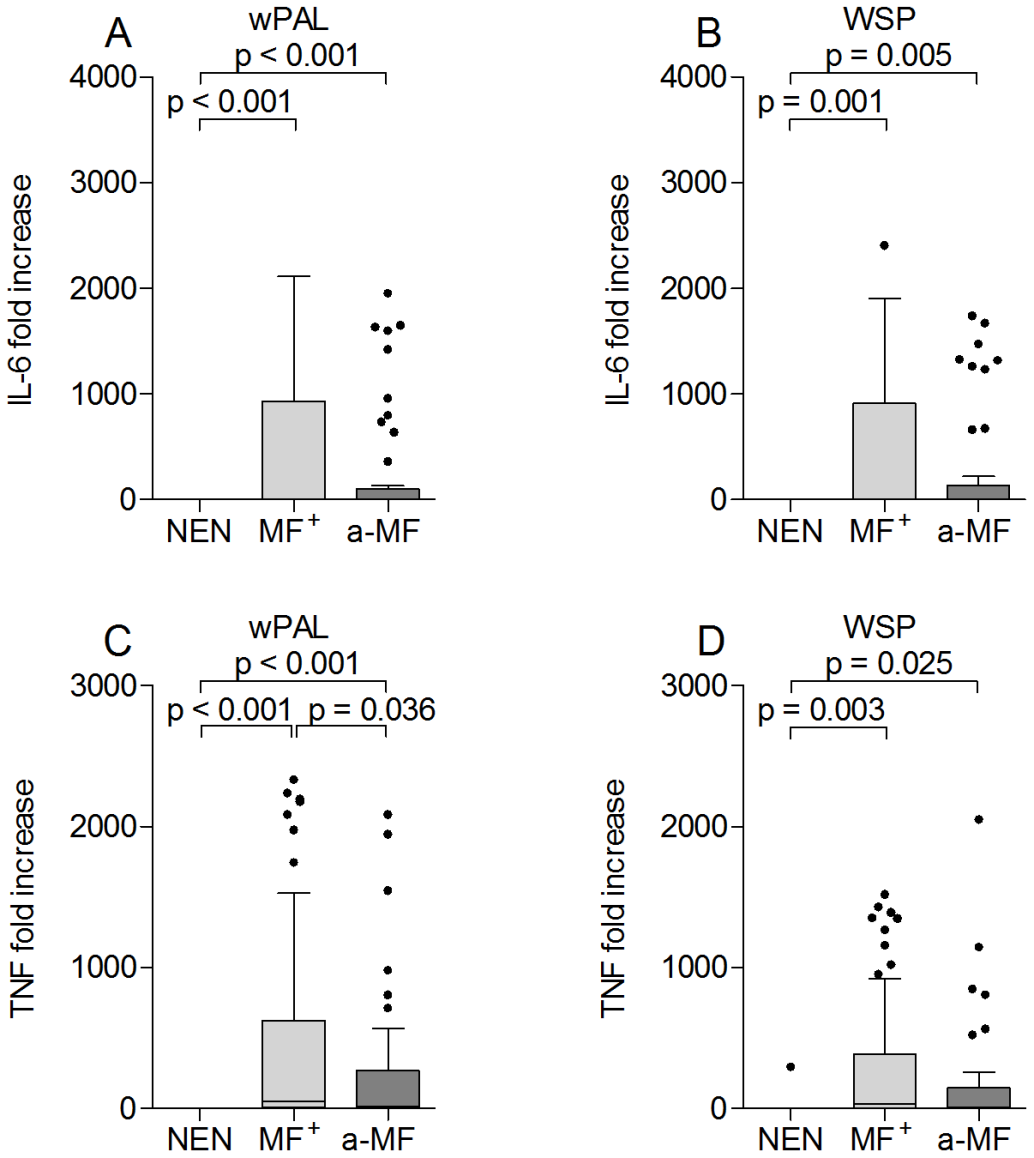
Induction of innate cytokines by *Wolbachia*-derived antigens. Isolated PBMCs (2×10^5^/well) from NEN or filarial infected MF^+^ or a-MF patients were stimulated with either wPAL (5 µg/ml) (A and C) or WSP (5 µg/ml) (B and D), for 72 hours. Thereafter, levels of IL-6 (A and B) and TNF (C and D) were measured in the culture supernatants via ELISA. Data are plotted as fold increase over unstimulated controls. Graphs show box whiskers with median, interquartile ranges and outliers. Statistical significances between the indicated groups were obtained after Kruskal-Wallis and Mann-Whitney tests.

After observing alterations in Th immune responses we addressed whether pro-inflammatory responses were also skewed in a-MF patients. Upon LPS stimulation infected groups showed significantly elevated levels of both IL-6 and TNF but no significant differences were observed between the two groups (Figures S4E and F). The same pattern was observed after measuring responses to PPD (Figures S3E and F). With regards to filarial-specific extracts, both infected groups showed elevated IL-6 responses following stimulation with OV and interestingly, these responses were significantly stronger than those elicited to BmFE and BmFEtet (Figure 6A), indicating that these reactions were mediated by components specific to *O. volvulus*. When compared to responses from NEN, TNF responses from both infected groups were also significantly elevated using OV or BmFE extracts (Figure 6B). Using the preparation of BmFEtet however, levels were no longer significant and PBMCs from NEN also began to respond (Figure 6B). Since these data indicated that the removal of *Wolbachia* decreased TNF responses we further analyzed PBMC responses using two *Wolbachia*-specific stimuli: Peptidoglycan-associated lipoprotein (wPAL) and *Wolbachia* surface protein (WSP). When compared to responses from NEN, PBMCs from infected patients produced elevated levels of IL-6 (Figures 7A and B) and TNF (Figures 7C and D) in response to wPAL and WSP. Moreover, when compared to responses from MF^+^ patients, a-MF individuals produced significantly less TNF in response to wPAL (Figure 7C).

### Multivariable regression analysis indicates that immune responses are associated with MF or ivermectin therapy on both the individual and community level

**Figure 8:**
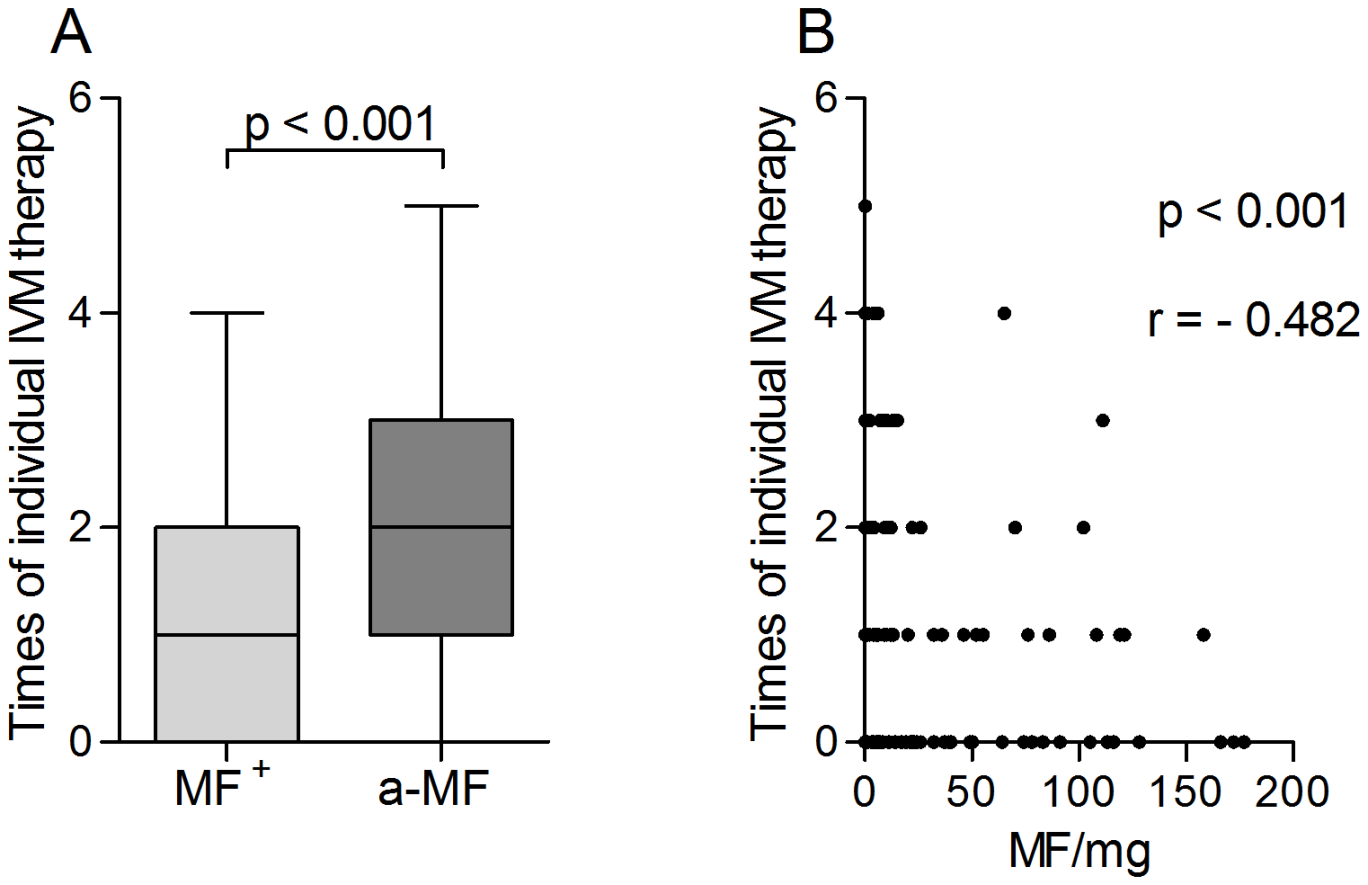
Individual IVM therapy is higher in a-MF^+^ individuals. Infected individuals completed a study questionnaire which included a section about the number of times they had taken IVM (IIT). The data are displayed as box whiskers with median, interquartile ranges and outliers (A). Statistical significances between the indicated groups were obtained after Mann-Whitney tests and significant differences are indicated in the figures. IIT was then correlated to the amount of skin residing microfilariae (B). Correlations were determined using the Spearman correlation test.

As described above, in many of the immunological parameters no significant differences could be observed between MF^+^ and a-MF individuals. However, a-MF patients did present a reduced amount of nodules (Figure 1) and decreased IL-10 responses to both filarial-specific and bystander stimuli (Figure 5, S3G, S4G). In addition, this group of individuals showed stronger IL-5 responses to OV antigens *in vitro* (Figure 4A) but reduced IL-5, eosinophils and ECP in plasma (Figure 3). The occurrence of a-MF patients is considered to be the result of repeated IVM treatment and/or missing re-infections [33]. Thus, we analyzed the number of individual IVM treatments (IIT) in our infection groups and as expected, a-MF patients had received IVM more often when compared to microfilaridermic patients (Figure 8A). Although age was increased in the former group (Figure S5A), there was no significant correlation between age and IIT (Figure S5B). However, there was a significant negative correlation between the amount of MF and times of individual IVM therapy (Figure 8B r = −0.482). To gain insight into whether the previous intake of ivermectin on an individual level or the frequency of IVM distribution within the community was influencing the immunological profile of a-MF patients we performed a multivariable regression analysis which included the variables “MF-positive”, “MF/mg”, “times of individual IVM therapy (IIT)”, “IVM in the last 12 months” and “Central∶Ashanti”. The latter refers to a regional-based covariate which consisted of 14 villages (82 individuals) from the “Central” region and 10 villages (128 individuals) from the “Ashanti” region. As mentioned above, at the time of sampling, MDA programmes had run 7 years longer in the Central region. Since our entire cohort had, on average, an individual IVM intake of 1.5 doses, any dominant associations between immune responses and the regional covariate indicate that this may result from the rounds of IVM within the community and in turn, lowered transmission rates. Table 2 shows a synopsis of the variables that were correlated with a particular immune response (right), and following multivariable analysis the factor that had the highest correlation (far right column). In many cases, the regression analysis supported the data shown in the above figures. For example, levels of IL-5 that were released upon re-stimulation with OV antigen had a negative correlation with MF/mg which confirms the differences that were observed between MF^+^ and a-MF individuals (Fig. 4B). Although not previously significant (Fig. 5B), IL-5 responses to Bm-derived antigens were also negatively correlated with MF/mg. In contrast and also in line with the immune profiles of a-MF patients was the result with *in situ* (plasma) levels of IL-5 since these results were highly associated with MF/mg (*c.f.* with Fig. 3D). Indeed, all *in situ* parameters were highly correlated to MF related covariates. Surprisingly, many IL-17 or pro-inflammatory responses were more associated with the regional covariate and in each situation the cytokine production of PBMCs isolated from individuals in the Central region was lower than those from patients in the Ashanti region. In more detail, with the exception of responses to OV antigen, all other IL-17 responses were associated with the region. This was also true for IL-6 responses apart from reactions to BmFEtet antigen which were negatively correlated to IIT instead. This covariate was also the most associated factor in the release of TNF to BmFEtet. Another novel association was the finding with WSP or WPAL antigens since these too were highly associated with the region and this was the only covariate to be associated with either TNF and IL-6 secretion to WSP. Finally, the most interesting outcome of the regression analysis was the finding with IL-10 responses. For example, whereas OV and BmFEtet responses were highly associated with IIT, BmFE responses were related to the presence of MF and αCD3/αCD28 and LPS responses were most strongly associated with the region. These results highlight the fact that the broad spectrum suppression of IL-10 responses in a-MF individuals was actually influenced by different factors.

**Table 2:** Summary of regression analysis between MF^+^ and a-MF patients.

Parameter	Stimulus	Associations with Univariable analysis	Multivariable analysis
	αCD3/αCD28	*MF/mg* *, *IVM 12 months* **	—
**IL-5**	OV	*MF/mg* **, *MF^+^* **, *IVM 12 months* **	MF/mg (neg. correlation, p<0.001)
	BmFE	*MF/mg* **, *MF^+^* *	MF/mg (neg. correlation, p = 0.017)
	BmFEtet	*MF/mg* **, *MF^+^* *	MF/mg(neg. correlation, p = 0.004)
**IL-13**	αCD3/αCD28	*MF/mg* **, *MF^+^* *	—
	αCD3/αCD28	*C∶A* **, *MF^+^* *, *IVM 12 months* **	C∶A (C↓A↑, p = 0.013)
	OV	*C∶A* **, *MF^+^* **, *IIT* **	IIT (neg. correlation, p = 0.011)
**IL-10**	BmFE	*MF^+^* **, *IIT* **	MF-positive (pos↑neg↓, p = 0.005)
	BmFEtet	*MF^+^* **, *IIT* **	IIT (neg. correlation, p = 0.003)
	PPD	*C∶A* *, *MF^+^* **, *IIT* *	—
	LPS	*C∶A* **, *MF^+^* *, *IIT* *	C∶A (C↓A↑, p = 0.016)
	OV	*C∶A* (C↓A↑, p = 0.001)	nd
	BmFEtet	*IIT* (neg. correlation, p = 0.014)	nd
**IL-6**	wPAL	*C∶A* **, *IVM 12 months* **	C∶A (C↓A↑, p<0.001)
	WSP	*C∶A*(C↓A↑, p = 0.007)	nd
	PPD	*C∶A* **, *IVM 12 months* **	C∶A (C↓A↑, p = 0.001)
	LPS	*C∶A* **, *MF^+^* *, *IVM 12 months* **	C∶A (C↓A↑, p = 0.015)
	BmFE	*IIT* **, *IVM 12 months* **	—
	BmFEtet	*IIT* (neg. Correlation, p = 0.02)	nd
**TNF**	wPAL	*C∶A* **, *MF^+^* **	C∶A (C↓A↑, p = 0.01)
	WSP	*C∶A*(C↓A↑, p = 0.038)	nd
	LPS	*C∶A* **, *MF^+^* *	—
	αCD3/αCD28	*C∶A* **, *MF/mg* **, *MF^+^* **, *IIT* **, *IVM 12 months* **	C∶A (C↓A↑, p<0.001)
**IL-17**	OV	*C∶A* *, *IIT* **	—
	BmFEtet	*C∶A* **, *IIT* *, *IVM 12 months* *	C∶A (C↓A↑, p = 0.01)
	PPD	*C∶A* **, *MF^+^* *	C∶A (C↓A↑, p = 0.017)
	LPS	*C∶A* **, *IIT* **, *IVM 12 months* **	C∶A (C↓A↑, p = 0.001)
**IFN-γ**	PPD	*MF-positive* (pos↓neg↑, p = 0.032)	
***In situ*** ** (plasma)**			
**IgE**		—	nd
**IgG4**		*C∶A*(C↓A↑, p = 0.036)	nd
**IL-5**		*MF/mg* **, *MF^+^* **, *IIT* **, *IVM 12 months* **	MF/mg (pos. correlation, p<0.001)
**Neutrophils**		*MF/mg* *, *MF^+^* **, *IIT* *	MF-positive (pos↓neg↑, p = 0.02)
**Eosinophils**		*MF/mg* **, *MF^+^* **, *IIT* **	MF/mg (pos. correlation, p<0.001)
**ECP**		*MF/mg* **, *MF^+^* **	MF/mg (pos. correlation, p<0.001)

C∶A denotes the Central∶Ashanti regional covariate. IIT denotes the number of times an individual had received IVM therapy.

* denotes p<0.1.

** denotes p<0.05.

‘nd’ denotes that only one covariate was below p<0.1 and therefore no multivariable analysis was done. ‘—’ denotes that no dominant factor was found in the multivariable analysis. Data were assessed using a generalized linear model analysis using the parameters “IIT”, “IVM intake within the last 12 months”, “MF-positivity”, “microfilarial density - Mf/mg” and “Central∶Ashanti” as covariates. The latter denotes a regional distribution covariate consisting of 14 villages (82 participants) in the Central region and 10 villages (128 individuals) in the Ashanti region. If more than one covariate had a p<0.1 following univariate analysis (depicted in italics), a further multivariable stage was conducted in order to identify the highest correlating factor.

## Discussion

After comparing 58 measured parameters, the main significant differences between MF^+^ and a-MF individuals were seen in classical hallmarks of *O. volvulus* infections: number of nodules, *in situ* (plasma) levels of IL-5, ECP and eosinophils and *O. volvulus*-specific IL-10 responses after stimulation *in vitro*. Moreover, using multivariable regression analysis our data suggests that MF or IVM therapy (on either an individual or community level) can differently influence certain immune parameters. Moreover, the data provides initial evidence that in response to diverse stimuli the secretion of a single cytokine (IL-10) from the same individual can be influenced by different factors. Of note, although both groups of patients displayed strong filarial-specific immunoglobulin levels no differences were observed. This is in agreement with other longitudinal studies that have shown no variations in Ig levels after several years of MDA [34]. Since both infected groups here were hyporesponsive (GEO), this result was somewhat anticipated and actually strengthens the differences that we and others have found between GEO and sowda patients. That is, high IL-10 and IgG4 in GEO individuals compared to high IgE, elevated Th2 responses and severe pathology in sowda patients [4], [9], [17], [29], [46]. This type of immune-regulation in GEO patients conforms with their increased worm burden and MF loads since high levels of IgG4 would counter-regulate IgE and therefore dampen overt responses to helminths [47]. Moreover, IgG4 is thought to aid MF survival by adhering to MF and preventing ADCC reactions elicited by other Ig subclasses and positive correlations were observed between IgG4 and MF in this study [48], [49]. To an extent the data from the regression analysis supports this hypothesis since there was a strong association of IgG4 with the region (Table 2) and as mentioned before, individuals from the Ashanti region had higher levels of MF than those residing in Central districts.

IL-10 is considered an immunosuppressive modulator acting on a number of innate and adaptive immune cells and deletion of the IL-10 gene can render mice more susceptible to infection pathology or autoimmune disease [50]. Humans display a substantial inter-individual variability and the intensity of IL-10 secretion has been linked to inherited polymorphisms [51]. Interestingly, Timman et al. determined that certain promoter haplotypes of IL-10 influence filarial-specific proliferation responses of PBMC from *O. volvulus* individuals [45] which correlates with studies showing that IL-10 neutralization reverses hypo-responsive T cells *ex vivo* [24]. Although such findings provide insight into genetic predispositions that could determine the outcome of infection, the analysis performed in our current study demonstrated that all infected patients produced more IL-10 upon classical T cell activation or filarial-specific re-stimulation. Interestingly, when compared to MF^+^ individuals, a-MF patients produced significantly less IL-10 to both filarial-specific and bystander stimuli (LPS and PPD). Moreover, following regression analysis it was revealed that IL-10 responses to different stimuli had varying correlations. For example, whereas responses to OV and BmFEtet had a negative correlation to IIT, responses to BmFE were highly associated with the presence of MF. Although the cellular source of IL-10 in filarial-infected patients remains undetermined it has been demonstrated that on average 75% of T cells, 10% B cells, 8% CD14^+^ monocytes and 7% NK cells produce IL-10 in response to filarial specific re-stimulation [52]. Moreover, even though a substantial proportion of these CD4^+^IL-10^+^ T cells made IL-4 but not IFN-γ, the majority produced neither [52] and this correlates with our earlier findings on T cell clones generated from subcutaneous tissue from patient nodules [20].

In contrast to the suppressed IL-10 responses, PBMCs from our a-MF infected cohort produced significantly higher levels of IL-5 to OV stimulus and this response was shown to be primarily associated with MF. Surprisingly however, when compared to MF^+^ individuals, circulating levels of IL-5 were significantly dampened in the a-MF group which was further substantiated in the multivariable regression analysis: positive correlation with MF/mg. Our a-MF cohort had viable adult worms and had received an average of only 2 rounds of IVM and perhaps therefore, their peripheral responses begin to reflect post patent individuals (+16 years of IVM therapy), which showed reduced filarial-specific IL-5 responses [31], [34]. This is the first study to determine opposing cytokine profiles *in situ* (plasma) and following stimulation *in vitro* and depicts how important it is to obtain an overall picture when trying to determine the immunomodulatory capacity of helminths. Reduced IL-5 levels in a-MF individuals were also reflected in the dampened percentage of eosinophils and amount of ECP but not neutrophils. In *O. volvulus* infections, eosinophil infiltration into the nodules is triggered by MF release and it has been shown that eosinophils actively attack MF. Indeed, Wildenburg et al. demonstrated that very few eosinophils were present in nodules that did not contain MF [53]. Eosinophils function through several factors; ECP for example, is primarily excreted by degranulating eosinophils and its secretion can be elicited in an either antibody-dependent or antibody-independent (complement) manner. Although its anti-helmintic properties remain unclear it is assumed that ECP works on MF rather than adult worms [54]. This hypothesis would support our findings that elevated levels of ECP were observed in MF^+^ but not a-MF individuals and all these peripherally-measured parameters were highly associated with the presence or density of MF. However, previous studies have shown that sowda patients have even higher levels of ECP than MF^+^ patients despite their low levels of MF [42]. In the studies performed here, IL-5, ECP and eosinophil number were all positively correlated in MF^+^ patients. Since these infection characteristics are also apparent in sowda patients [42] we conclude that a-MF patients have a unique immune profile in which neutrophils play a prominent role.

Neutrophils have been a rather neglected cell population but numerous pieces of research are beginning to reveal a see-saw relationship between eosinophils and neutrophils during filarial infections. Studies have shown that both eosinophils and neutrophils increased shortly after IVM therapy with the former being strongly associated with MF elimination within the draining lymph nodes [55], [56]. Our data shows that neutrophils are significantly elevated in a-MF patients even after a lengthy interval of individual IVM therapy but according to our regression analysis it is the presence of MF that is most associated with neutrophil numbers. Peripheral eosinophils were strongly down-regulated in a-MF individuals and multivariable regression analysis revealed that this immune factor had a positive correlation with the density of MF in the skin. Interestingly, it appears that nodule-forming *Onchocerca* spp. can coexist with large numbers of infiltrating neutrophils for many years without any apparent impediment to the parasites' physiology or reproduction and moreover, such cells may even protect the worm from eosinophil degranulation. As mentioned above, *O. volvulus* worms depend on their endosymbiotic relationship with *Wolbachia* which reside within the lateral cords and reproductive apparatus [16]. Indeed, this essential partnership has provided alternative chemotherapeutic approaches since elimination of *Wolbachia* by antibiotic treatment causes sterility and eventually worm death [14], [57]. It has been shown that neutrophils are attracted to *Wolbachia*-derived molecules [58], but following antibiotic chemotherapy they are replaced by eosinophils that degranulate on the worm cuticle. In a study with *O. ochengi*, the filarial parasite of cattle, eosinophil degranulation was significantly increased following antibiotic but not conventional adulticidal regimes and was associated with worm vitality not degeneration [41]. Nevertheless, it remains unclear during *O. volvulus* infections whether the influx of eosinophils is an intentional act to kill the parasite and moreover, what parasitic or immune scenario they are attracted to.

Much research has focused on elucidating *Wolbachia*-derived components that immunomodulate host immune responses. WSP is a highly conserved *Wolbachia* surface protein and has been noted to stimulate innate responses through distinctive TLR family members [4], [38]. WSP has been shown to induce the release of IL-8 from human neutrophils and has anti-apoptotic effects that are not mediated through Fas-pathways [59]. Here, we found that preparations of WSP and wPAL elicited IL-6 and TNF from infected patients and moreover, TNF levels were reduced in a-MF individuals. Furthermore, levels of TNF secretion from infected individuals were reduced when cells were exposed to worm preparations depleted of *Wolbachia*. Collectively these data indicate that a-MF patients have reduced *Wolbachia*-specific responses. Similarly, when compared to MF^+^ individuals, responses to innate stimuli (LPS) were also weaker in a-MF patients although both groups of infected patients responded stronger than NEN. The regression analysis revealed that TNF and IL-6 responses to wPAL were highly associated with regional distribution and with regards to WSP this was the only associated covariate. Perhaps such modulations are related to longer previous IVM MDA in the Central Region and consequently lower transmission and less exposure to incoming L3. Since the vast majority of L3 are cleared by the human host, as a consequence there might be less *Wolbachia*-derived signals from perishing L3, which decreases the responsiveness to *Wolbachia*. Alternatively the reduced MF load following IVM in turn decreases the exposure of *Wolbachia*-derived signals and therefore the immunomodulatory capacity of such signals is also reduced.

As with other research on the immune status of *O. volvulus*-infected individuals we found very little activity in terms of IFN-γ which was confirmed by the overall lack of association(s) in the regression analysis. Interestingly, although there were no significant differences in IL-17 responses between MF^+^ and a-MF individuals, the regression analysis revealed multiple associations with the regional covariate. For example, with the exception OV antigen (equally associated with IIT and region), all other IL-17 responses were highly associated with the region. Since IL-17 levels were lower in individuals from the Central region and moreover people residing in that region had had a higher intake of IVM in the last 12 months, it indicates that MDA therapy generates less IL-17 responses or the presence of MF dampens them. With regards to IL-13, our data partially substantiates the findings of Brattig et al. [60], since levels were significantly elevated in infected groups upon αCD3/αCD28 stimulation and this response was strongly related to the presence of MF. Although this indicates an elevated Th2 profile, no differences could be observed between the groups when cells were exposed to either filarial-specific or bystander stimuli. However, when comparing the responses of MF^+^ patients to the different filarial antigens, responses to *Wolbachia* depleted antigens were significantly lower. Differences in immune responses between studies may not only lie in how helminth antigen extracts are prepared but also in the experimental study design (whole blood assay versus isolated PBMC re-stimulation; since cell concentrations may vary in the former) or even the amount of antigen added to the cultures and such aspects require further investigation.

Alongside their unique immune status, a-MF patients also presented a reduced amount of palpable nodules and sites and this parameter positively correlated to the number of MF but not the intake of ivermectin on an individual level (IIT). Introducing a regional covariate revealed a surprising number of associations especially IL-17, IL-6 and TNF secretions. Indeed, for some immune scenarios such as levels of IL-6 in response to OV antigen or IL-6 and TNF responses to WSP this was the only associated factor. Moreover, in each situation the cytokine production of PBMCs isolated from individuals in the Central region was lower than those from patients in the Ashanti region. Although an a-MF status in adults may occur in individuals with single sex infections, in pre-patency or in older patients with aged infertile females, the experimental data and regression analysis shown here strongly indicates that these distinct immune profiles stem from three covariates: MF, IIT and the frequency of IVM distribution within the community. Moreover, the analysis reveals that the release of different cytokines by an individual to a certain stimuli can be dependent on different factors. In conclusion, despite our cohort having an average individual IVM intake of 1.5, highly significant associations with the region or frequency of MDA within the community suggest that a lowered infection pressure due to IVM MDA may affect community members that have not regularly participated in MDA programmes, implying that immune responses are affected by the number of times an individual had received IVM *and* the amount of MDA therapy on a community level. Perhaps such details will help to understand IVM (immune-) mediated MF killing and also suboptimal performances of IVM regarding this activity.

## Supporting Information

Figure S1 **Correlation of neutrophils and eosinophils with MF.** Percentages of eosinophils and neutrophils from infected individuals were either correlated to each other (A) or to the amount of skin microfilariae (B and C respectively). Correlations were determined using the Spearman correlation test.
(TIF)

Figure S2 **Filarial antigens induce no alterations in IL-17 or IFN-γ responses.** Isolated PBMCs (2×10^5^/well) from NEN or *O. volvulus* infected MF^+^ or a-MF patients were stimulated with either anti-CD3/anti-CD28 (A and E), OV (B and F), BmFE extract (C and G) or BmFEtet extract (D and H) for 72 hours. Thereafter, levels of IL-17 (A–D) and IFN-γ (E–H) were measured in the culture supernatants via ELISA. Data are plotted as fold increase over unstimulated controls. Graphs show box whiskers with median, interquartile ranges and outliers. Statistical significances between the indicated groups were obtained after Kruskal-Wallis and Mann-Whitney tests.
(TIF)

Figure S3 **PPD responses in** ***O. volvulus*** **exposed individuals.** Isolated PBMCs (2×10^5^/well) from NEN or *O. volvulus* infected MF^+^ or a-MF patients were stimulated with PPD (10 µg/ml) for 72 hours. Thereafter, levels of IL-5 (A), IL-13 (B), IL-17 (C), IFN-γ (D), IL-6 (E), TNF (F) and IL-10 (G) were measured in the culture supernatants via ELISA. Data are plotted as fold increase over unstimulated controls. Graphs show box whiskers with median, interquartile ranges and outliers. Statistical significances between the indicated groups were obtained after Kruskal-Wallis and Mann-Whitney tests.
(TIF)

Figure S4 **Elevated TLR-triggered responses in infected individuals.** Isolated PBMCs (2×10^5^/well) from NEN or *O. volvulus* infected MF^+^ or a-MF patients were stimulated with LPS (50 ng/ml) for 72 hours. Thereafter, levels of IL-5 (A), IL-13 (B), IL-17 (C), IFN- γ (D), IL-6 (E), TNF (F) and IL-10 (G) were measured in the culture supernatants via ELISA. Data are plotted as fold increase over unstimulated controls. Graphs show box whiskers with median, interquartile ranges and outliers. Statistical significances between the indicated groups were obtained after Kruskal-Wallis and Mann-Whitney tests.
(TIF)

Figure S5 **Relating age and individual IVM therapy (IIT) in a-MF patients.** Within the questionnaire all *O. volvulus* infected individuals were asked for their age (A). Graph shows a scatter plot with median. Statistical significances between the groups were obtained after Mann-Whitney tests. The number of times an individual had taken IVM therapy (IIT) was then correlated to age (B).
(TIF)
