# Supplementary figures and images for: Immunoepidemiological Profiling of Onchocerciasis Patients Reveals Associations with Microfilaria Loads and Ivermectin Intake on Both Individual and Community Levels
Source: PLoS Negl Trop Dis. 2014 Feb 20;8(2):e2679. doi: 10.1371/journal.pntd.0002679 (PMC3930501; doi:10.1371/journal.pntd.0002679)

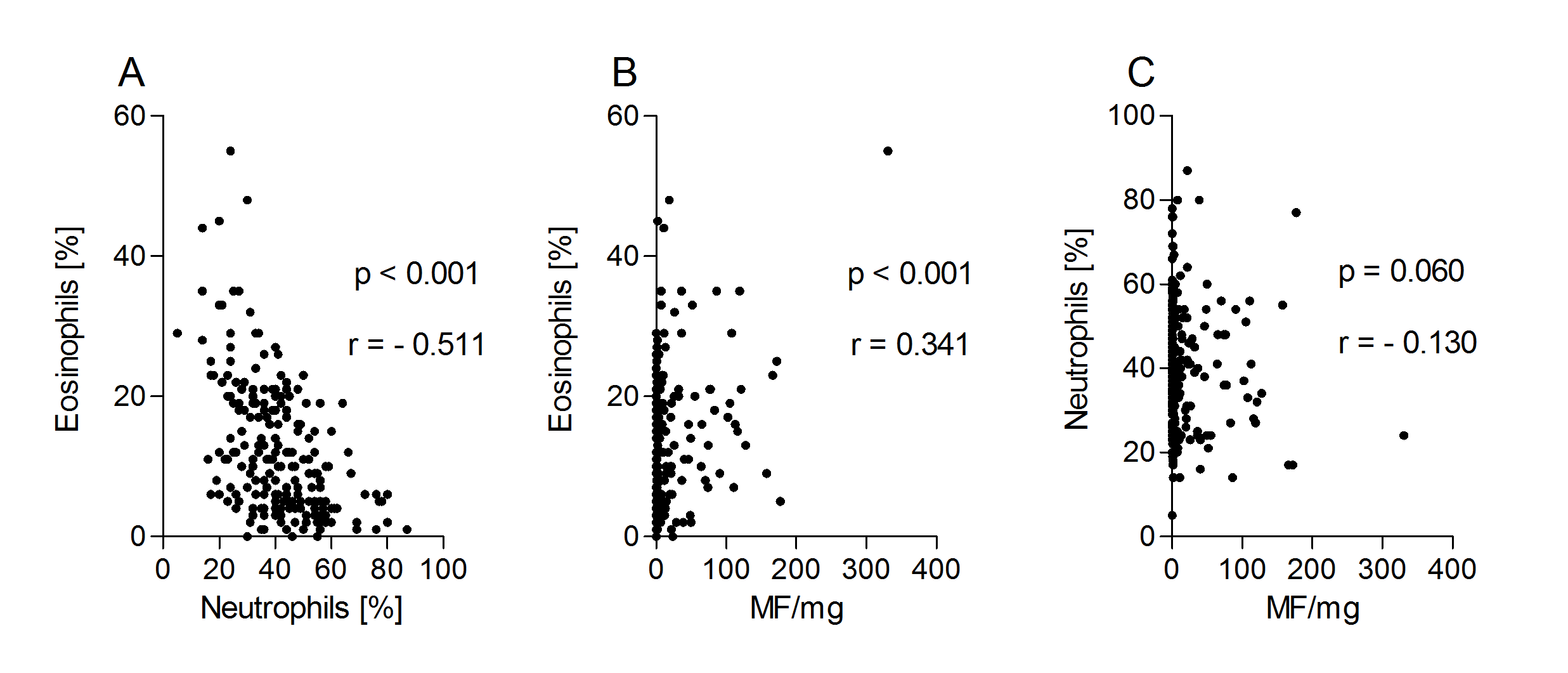

Supplement: Figure S1 — Correlation of neutrophils and eosinophils with MF. Percentages of eosinophils and neutrophils from infected individuals were either correlated to each other (A) or to the amount of skin microfilariae (B and C respectively). Correlations were determined using the Spearman correlation test. (TIF) [file pntd.0002679.s001.tif]

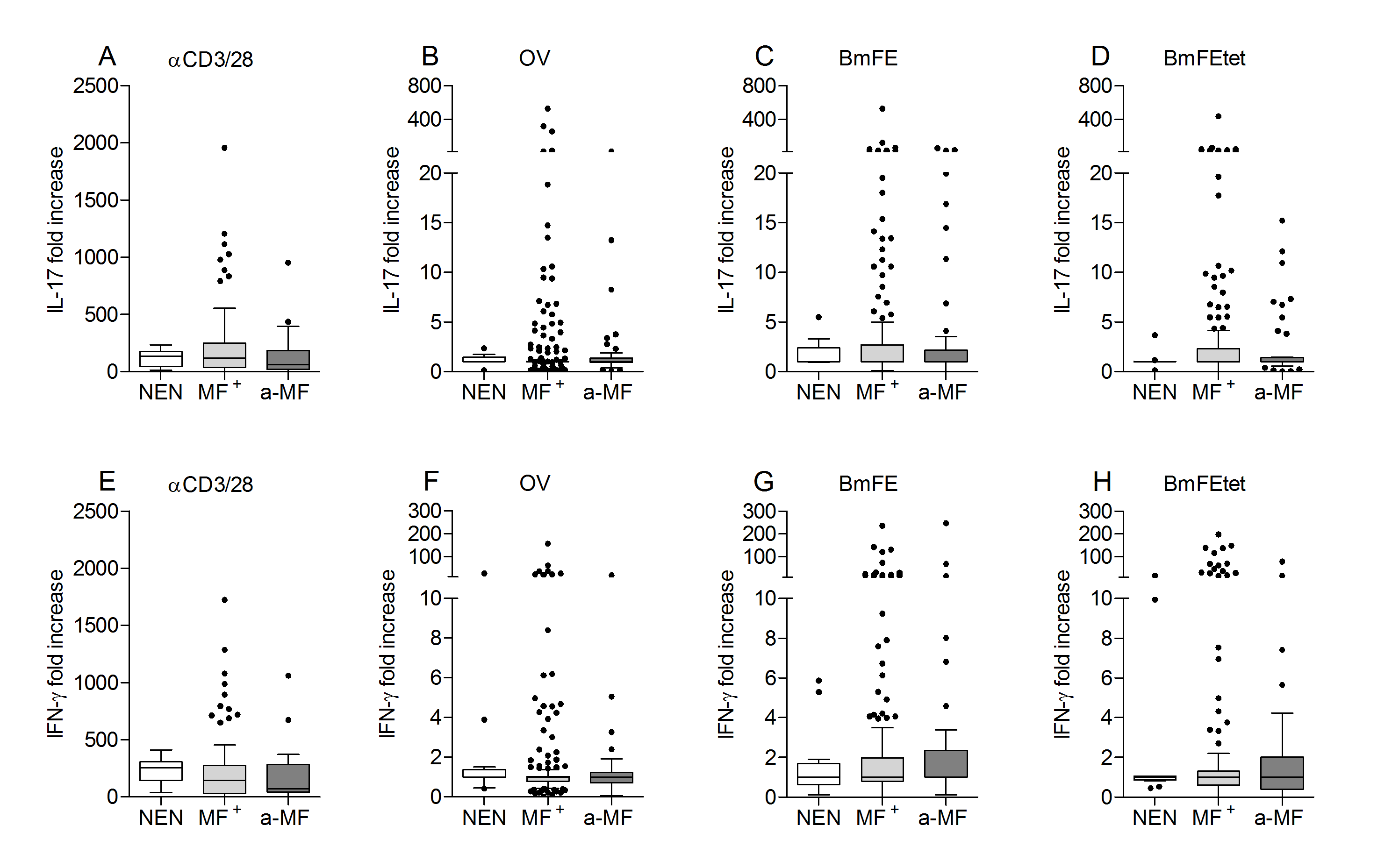

Supplement: Figure S2 — Filarial antigens induce no alterations in IL-17 or IFN-γ responses. Isolated PBMCs (2×10^5^/well) from NEN or O. volvulus infected MF+ or a-MF patients were stimulated with either anti-CD3/anti-CD28 (A and E), OV (B and F), BmFE extract (C and G) or BmFEtet extract (D and H) for 72 hours. Thereafter, levels of IL-17 (A–D) and IFN-γ (E–H) were measured in the culture supernatants via ELISA. Data are plotted as fold increase over unstimulated controls. Graphs show box whiskers with median, interquartile ranges and outliers. Statistical significances between the indicated groups were obtained after Kruskal-Wallis and Mann-Whitney tests. (TIF) [file pntd.0002679.s002.tif]

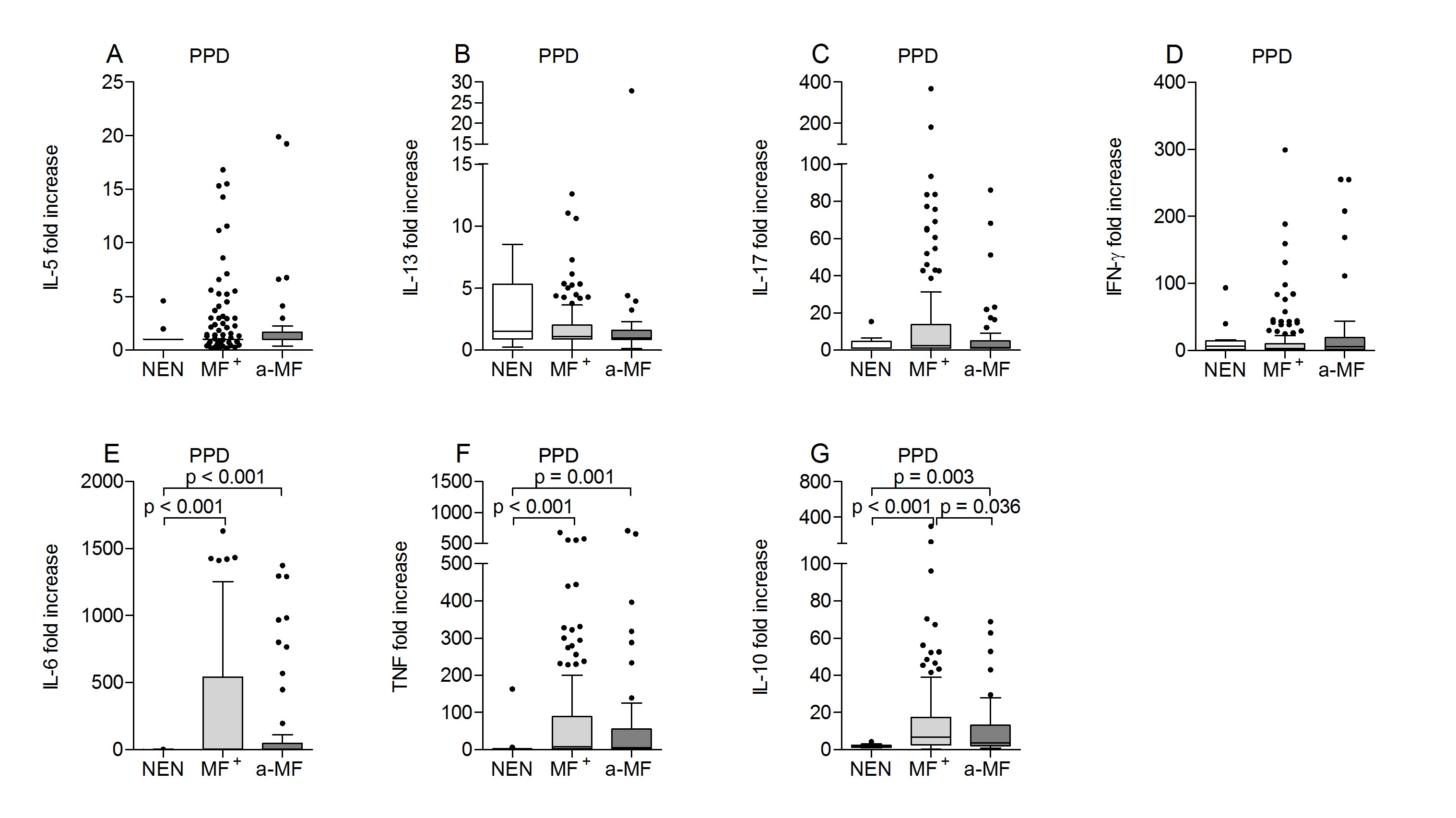

Supplement: Figure S3 — PPD responses in O. volvulus exposed individuals. Isolated PBMCs (2×10^5^/well) from NEN or O. volvulus infected MF+ or a-MF patients were stimulated with PPD (10 µg/ml) for 72 hours. Thereafter, levels of IL-5 (A), IL-13 (B), IL-17 (C), IFN-γ (D), IL-6 (E), TNF (F) and IL-10 (G) were measured in the culture supernatants via ELISA. Data are plotted as fold increase over unstimulated controls. Graphs show box whiskers with median, interquartile ranges and outliers. Statistical significances between the indicated groups were obtained after Kruskal-Wallis and Mann-Whitney tests. (TIF) [file pntd.0002679.s003.tif]

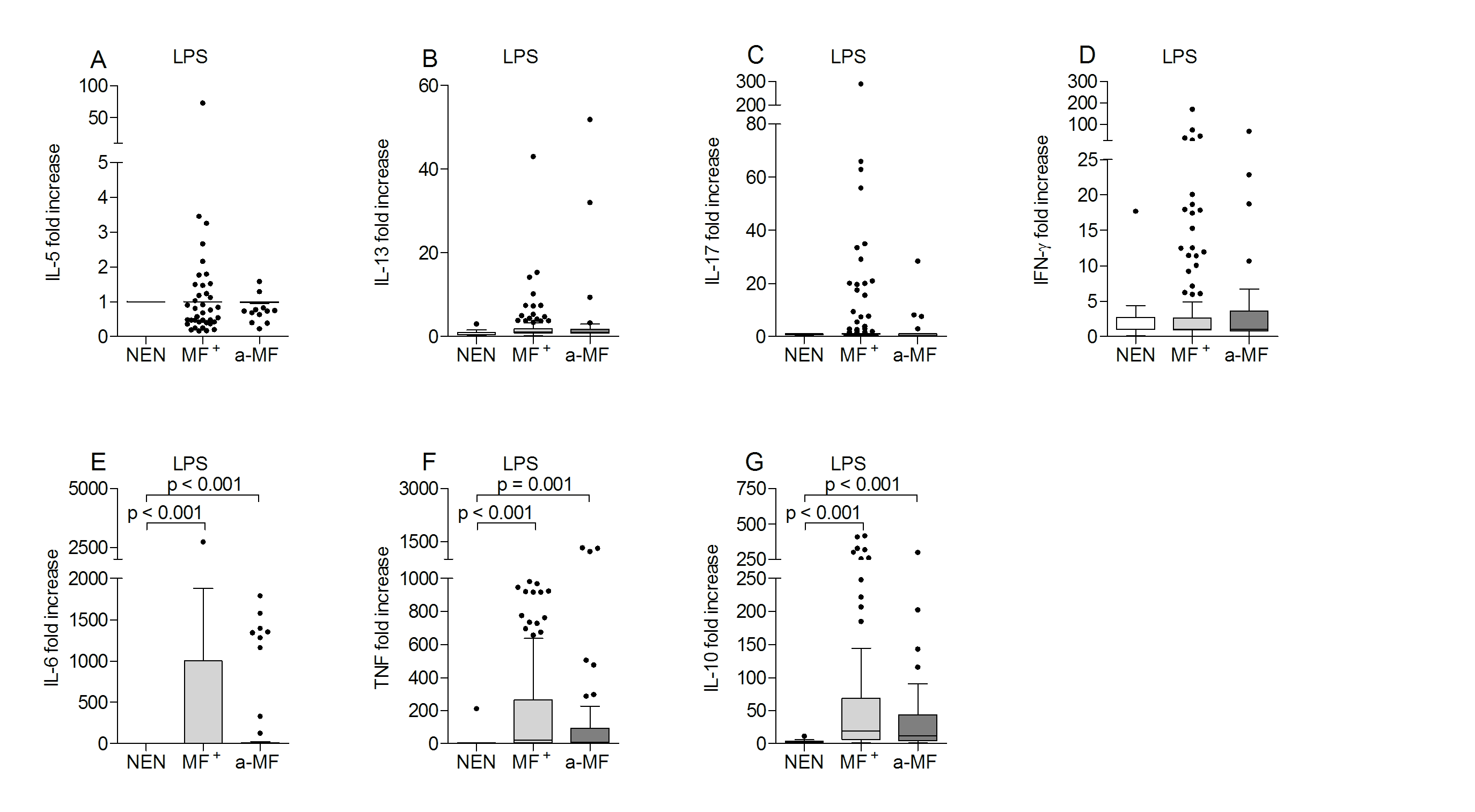

Supplement: Figure S4 — Elevated TLR-triggered responses in infected individuals. Isolated PBMCs (2×10^5^/well) from NEN or O. volvulus infected MF+ or a-MF patients were stimulated with LPS (50 ng/ml) for 72 hours. Thereafter, levels of IL-5 (A), IL-13 (B), IL-17 (C), IFN- γ (D), IL-6 (E), TNF (F) and IL-10 (G) were measured in the culture supernatants via ELISA. Data are plotted as fold increase over unstimulated controls. Graphs show box whiskers with median, interquartile ranges and outliers. Statistical significances between the indicated groups were obtained after Kruskal-Wallis and Mann-Whitney tests. (TIF) [file pntd.0002679.s004.tif]

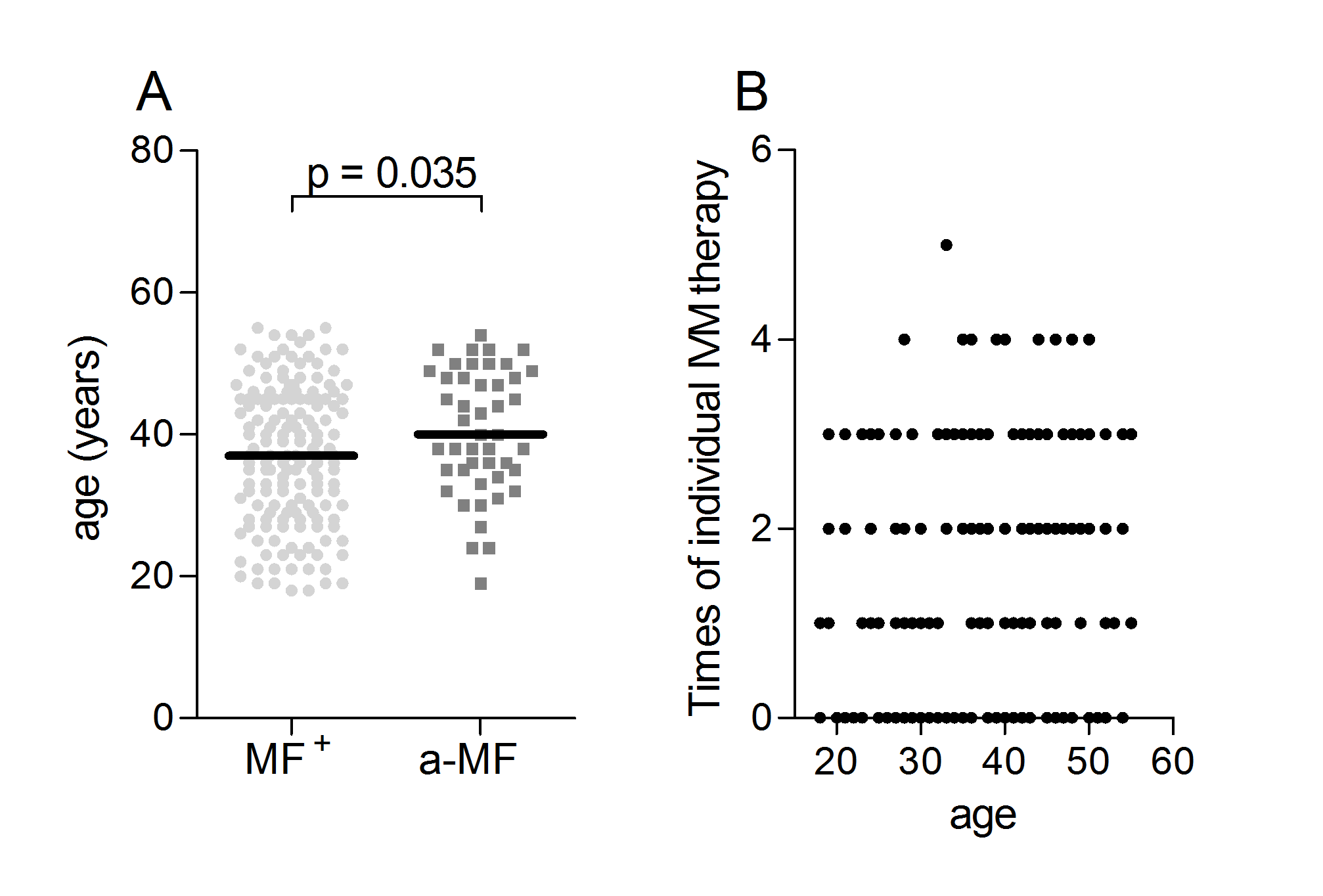

Supplement: Figure S5 — Relating age and individual IVM therapy (IIT) in a-MF patients. Within the questionnaire all O. volvulus infected individuals were asked for their age (A). Graph shows a scatter plot with median. Statistical significances between the groups were obtained after Mann-Whitney tests. The number of times an individual had taken IVM therapy (IIT) was then correlated to age (B). (TIF) [file pntd.0002679.s005.tif]
